# BOW-GBDT: A GBDT Classifier Combining With Artificial Neural Network for Identifying GPCR–Drug Interaction Based on Wordbook Learning From Sequences

**DOI:** 10.3389/fcell.2020.623858

**Published:** 2021-02-01

**Authors:** Wangren Qiu, Zhe Lv, Yaoqiu Hong, Jianhua Jia, Xuan Xiao

**Affiliations:** ^1^School of Information Engineering, Jingdezhen Ceramic Institute, Jingdezhen, China; ^2^School of Information Engineering, Jingdezhen University, Jingdezhen, China

**Keywords:** GPCR-drug interaction, bag-of-words, weighted silhouette coefficient, discrete wavelet transform, artificial neural network

## Abstract

**Background:** As a class of membrane protein receptors, G protein-coupled receptors (GPCRs) are very important for cells to complete normal life function and have been proven to be a major drug target for widespread clinical application. Hence, it is of great significance to find GPCR targets that interact with drugs in the process of drug development. However, identifying the interaction of the GPCR–drug pairs by experimental methods is very expensive and time-consuming on a large scale. As more and more database about GPCR–drug pairs are opened, it is viable to develop machine learning models to accurately predict whether there is an interaction existing in a GPCR–drug pair.

**Methods:** In this paper, the proposed model aims to improve the accuracy of predicting the interactions of GPCR–drug pairs. For GPCRs, the work extracts protein sequence features based on a novel bag-of-words (BOW) model improved with weighted Silhouette Coefficient and has been confirmed that it can extract more pattern information and limit the dimension of feature. For drug molecules, discrete wavelet transform (DWT) is used to extract features from the original molecular fingerprints. Subsequently, the above-mentioned two types of features are contacted, and SMOTE algorithm is selected to balance the training dataset. Then, artificial neural network is used to extract features further. Finally, a gradient boosting decision tree (GBDT) model is trained with the selected features. In this paper, the proposed model is named as BOW-GBDT.

**Results:** D92M and Check390 are selected for testing BOW-GBDT. D92M is used for a cross-validation dataset which contains 635 interactive GPCR–drug pairs and 1,225 non-interactive pairs. Check390 is used for an independent test dataset which consists of 130 interactive GPCR–drug pairs and 260 non-interactive GPCR–drug pairs, and each element in Check390 cannot be found in D92M. According to the results, the proposed model has a better performance in generation ability compared with the existing machine learning models.

**Conclusion:** The proposed predictor improves the accuracy of the interactions of GPCR–drug pairs. In order to facilitate more researchers to use the BOW-GBDT, the predictor has been settled into a brand-new server, which is available at http://www.jci-bioinfo.cn/bowgbdt.

## Background

As a special membrane protein, G protein-coupled receptors (GPCRs) play a significant role in the normal life function of cells (Jacoby et al., 2006) and can be used as important drug targets because of its structural characteristics and important role in signal transduction (Agrawal et al., 2016). Among the most popular drugs in the market, nearly half of them work through GPCRs directly or indirectly (Alexander et al., 2011). Therefore, it is of much significance to find GPCRs that interact with drugs in the process of drug development (Alberts et al., 2003; Alexander et al., 2011).

High-throughput experimental methods such as scintillation proximity assay and time-resolved fluorescence resonance energy transfer technology are the key in GPCR-related drug discovery (Zhang and Xie, 2012). However, experimental methods are inevitably costly, labor-exhausting, and time-consuming. As predicting the interaction of GPCR–drug pairs will help to avoid wasting a lot of time and money in synthetic drug research, prediction approaches *in silico* are widely utilized to assist the experimental methods with the rapid development of prediction algorithms and datasets.

In recent years, a number of researchers have proposed effective predicted methods which are based on 3D structures of GPCR for predicting the target drug interaction (Yamanishi et al., 2008; Ru et al., 2020). However, a lot of 3D structures of GPCR have not been measured yet. As a result, the application of these methods based on the 3D structures of proteins is greatly restricted. With the accumulation of GPCR–drug interaction data stored in Kyoto Encyclopedia of Genes and Genomes (KEGG) (Kanehisa et al., 2006), SuperTarget (Gunther et al., 2008), and DrugBank (Wishart et al., 2008), methods based on sequence information may be efficient for identifying the interaction between GPCR and drug. Therefore, we will focus the research only based on sequence information in this study.

Since the interaction between GPCRs and drugs involves two types of molecules, the method which combines the chemical structure information of drugs and the sequence information of proteins is often used. Yamanishi et al. (2008) used statistical methods to predict the GPCR–drug interaction based on the combination of protein chemical structure and sequence information. On the basis of optimizing the feature selection process, He et al. (2010) used the nearest neighbor algorithm as a classifier to predict the interaction between drugs and four targets including GPCRs. In this method, drug was formulated into a 28-D vector based on the chemical functional group, and protein was formulated into a 139-D vector using a pseudo-amino acid composition (PseAAC) (Arif et al., 2018; Mei and Zhao, 2018). Xiao et al. (2013) proposed a sequence-based predictor called “iGPCR–drug”. In the predictor, the component of the drug was represented by a two-dimensional fingerprint *via* a chemical toolbox called OpenBabel (O'Boyle et al., 2011), and then discrete Fourier transform (DFT) was used to extract 256 features. The GPCR was composed of PseAAC generated with the gray model theory, and the prediction engine adopted the fuzzy *K*-nearest neighbor algorithm. TargetGDrug (Hu et al., 2016) was also a sequence-based predictor for predicting GPCR–drug interactions. The method formed the features of the GPCR–drug pair by combining the evolutionary features of the GPCR sequence with the molecular fingerprint features of the drug based on discrete wavelet transform and input the features into a trained random forest classifier for initial prediction. Finally, a new post-processing procedure based on drug association matrix is proposed to reduce potential false positives or false negatives in initial predictions. Recently, Wang et al. (2020b) proposed a novel sequence-based method for identifying the GPCR–drug interaction. In such work, the sequences of GPCRs were encoded by the physicochemical properties of amino acids, and then clustering technology was used to create four wordbooks. The wordbooks contained 20, 20, 30, and 58 words which are determined with the method of trial and error; it is a little tedious and unreliable. Then, the GPCR–drug pairs were concatenated to a 256-D vector comprising of a 128-D wordbooks vector for GPCR and a 128-D DFT vector for drugs with fingerprint. Finally, a simple machine learning algorithm, distance-weighted *K*-nearest neighbors (DWKNN) (Dudani, 1976), was adopted as the predictor *via* training on eventual features. Although this advanced model was better than the foregoing ones, the machine learning algorithm was such simple that it could not get a better performance, so it is meaningful to employ advanced algorithm to develop a model with higher performance.

In this study, we propose a novel sequence-based machine learning model for identifying the GPCR–drug interaction based on wordbook learning from sequences. For GPCR, we use an improved bag-of-words (BOW) (Wang et al., 2020b) model containing four wordbooks to extract features by introducing silhouette coefficient to determine the best number of words. For the drug, we carry out discrete wavelet transform (DWT) on molecular fingerprint to extract features. The SMOTE algorithm is implemented to balance the training dataset, and an artificial neural network (ANN) (Rumelhart et al., 1986; Hinton and Salakhutdinov, 2006; Hinton, 2007; Zou et al., 2016; Wan et al., 2017; Chao et al., 2019) model is used to extract GPCR–drug pair features and reduce the dimension from 242-D to 121-D. A more effective algorithm called gradient boosting decision tree (GBDT) (Friedman, 2001; Lv et al., 2020a; Sahin, 2020) is employed as the classifier for interaction prediction. According to the result on the independent test dataset, the proposed model, BOW-GBDT, can achieve better performance than those of the existing references.

## Datasets and Methods

### Experimental Datasets and Performance Measurement

In this study, two benchmark datasets, i.e., D92M and Check390 (Hu et al., 2016), are served for testing the proposed method. D92M is used for a cross-validation dataset which contains 635 interactive GPCR–drug pairs and 1,225 non-interactive pairs. Check390 is used for an independent test dataset which consists of 130 interactive GPCR–drug pairs and 260 non-interactive GPCR–drug pairs, and each element in Check390 cannot be found in D92M. In our experiment, we evaluate the performance of the predictor from five metrics listed in formula (1), which include accuracy (Acc), sensitivity (Sn), specificity (Sp), Matthews correlation coefficient (MCC), and strength (Str, the average of Sn and Sp) (Cheng et al., 2019). In the following formula, TP is the number of the actual interactive GPCR–drug pairs predicted as interactive GPCR–drug pairs, TN is the number of the actual non-interactive pairs predicted as non-interactive pairs, FP is the number of the actual non-interactive pairs but predicted as interactive pairs, and FN is the number of the actual interactive pairs but predicted as non-interactive pairs. What is more, receiver operating characteristic (ROC) curve and area under the ROC curve (AUC) are also applied to evaluate the models in this work.

(1) $$\begin{array}{l} \left\{ \begin{array}{c} \text{Accuracy}=\frac{\text{TP}+\text{TN}}{\text{TP}+\text{TN}+\text{FP}+\text{FN}}\\ \text{Sensitivity}=\frac{\text{TP}}{\text{TP}+\text{FN}}\\ \text{Specificity}=\frac{\text{TN}}{\text{TN}+\text{FP}}\\ \text{Strength}=\frac{\text{Sensitivity}+\text{Specificity}}{2}\\ \text{MCC}=\frac{\text{TP}\times \text{TN}-\text{FP}\times \text{FN}}{\sqrt{\left(\text{TP}+\text{FP}\right)\left(\text{TP}+\text{FN}\right)+\left(\text{TN}+\text{FP}\right)\left(\text{TN}+\text{FN}\right)}} \end{array}\right. \end{array}$$

### Feature Extraction From GPCRs

Based on the work of Wang et al. (2020b), who developed an effective method to represent GPCR with BOW model, this study will enhance the BOW model by using weighted silhouette coefficient and a variety of ways for determining the wordbooks. The steps of feature extraction are as follows:

- Step 1: Encoding GPCRs with amino acid index

AAindex (Kawashima and Kanehisa, 2000) is a database which collects more than 500 amino acid indices. Wang et al. (2020b) tested the effects of five common amino acid indices: hydropathy index, molecular weight, isoelectric point, pK-N, and pK-C. According to the experimental result, we choose hydropathy index as the suitable amino acid index in this paper.

- Step 2: Designing wordbooks for GPCRs

In this paper, four kinds of wordbooks were defined and denoted as *WB*_*A*_, *WB*_*B*_, *WB*_*C*_, and *WB*_*D*_. GPCR sequences were encoded according to hydropathy index. Here the amino acid composition (AAC) is the candidate for wordbook *WB*_*A*_, which is the same as in Wang et al. (2020b), and the number of words is 20 obviously.

**Figure 1:**
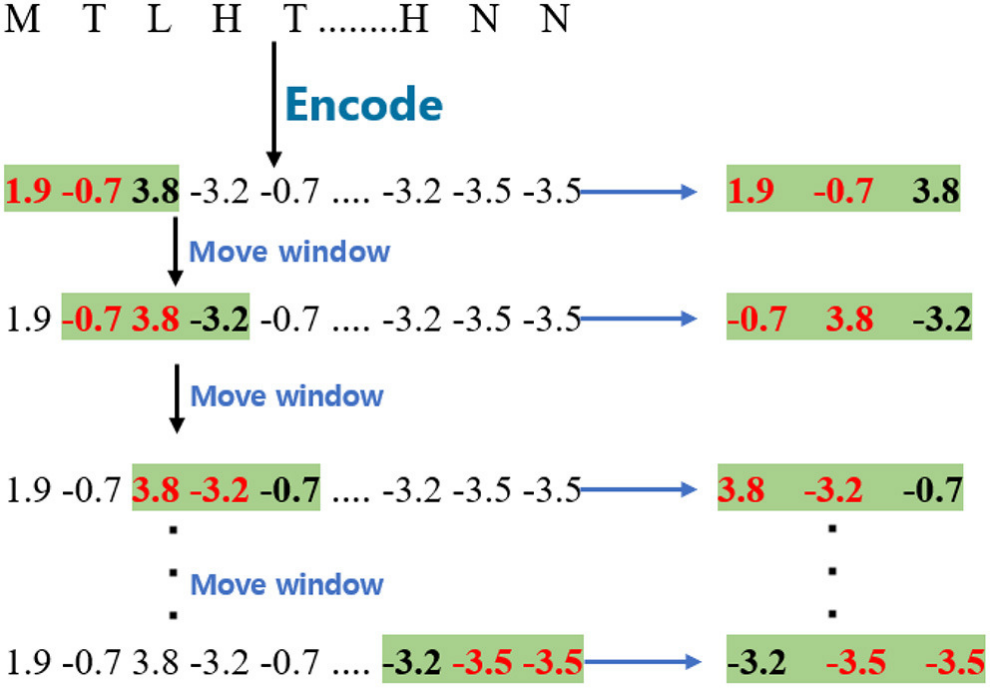
The process of cutting G protein-coupled receptor sequence for *WB*_*B*_ and *WB*_*C*_.

To obtain wordbook *WB*_*B*_, the encoded sequences were split into fragments with different window sizes. The window size of *WB*_*B*_ was set as 2, and the stride of the moving window is 1 in this paper. Given a GPCR sequence, the process of this step is shown in Figure 1. The window size is 2 in the example.

As regards wordbook *WB*_*C*_, the model applies a similar process to obtain them except that the window size is 3, and the process of cutting the GPCR sequence is marked with a green background as shown in Figure 1.

**Figure 2:**
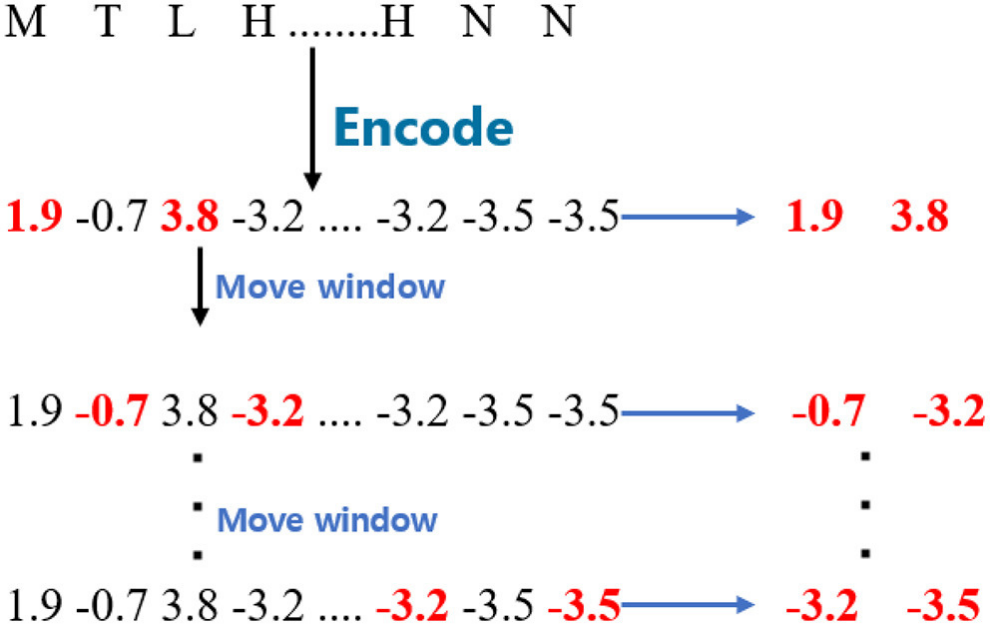
The process of cutting G protein-coupled receptor sequences for *WB*_*D*_.

For the *WB*_*D*_, the proposed model split the encoded sequences into fragments with a window size 2. The window is different from the one used in *WB*_*B*_ and *WB*_*C*_ since it is separated by one amino acid. The stride of the moving window is also 1. Given a GPCR sequence, the process is shown in Figure 2.

- Step 3: Determine the best number of the clustering by using weighted silhouette coefficient

Here we used *K*-means (Hartigan and Wong, 1979; Kanungo et al., 2002) algorithm to cluster the fragments with the same length, respectively, and take the clustering centers as the words of the GPCR wordbook. In the process of creating the four kinds of wordbooks, it is important to determine the numbers of the clustering centers which would fluctuate the results greatly (Wang et al., 2020b). In this step, a metric called weighted silhouette coefficient (Rousseeuw, 1987) (noted as WSC, an evaluation method of clustering effect) was used to decide the best *K* which is the number of clustering centers.

For a given dataset, *D* = {(*x*_1_, *y*_1_), (*x*_2_, *y*_2_), ⋯, (*x*_*M*_, *y*_*M*_)}, *y*_*i*_ is the label of the sample *x*_*i*_, *y*_*i*_ ∈ {1, ⋯, *C*}, and *C* is the number of clusters. The weighted silhouette coefficient would be calculated with the formula $WSC=\;\frac{\overset{M}{\underset{i=1}{\sum }}WSC_{x_{i}}}{M}$ according to Rousseeuw (1987), in which *WSC*_*x*_*i*__ is the WSC of sample *x*_*i*_ and obtained with the following steps:

Firstly, for any sample *x*_*i*_, *D*_*x*_*i*__ = {(*x, y*)|(*x, y*) ∈ *D and y* = *y*_*i*_}, let ${\bar{d}}_{x_{i}}^{in}$ represents the internal means distance which can be obtained with formula (2)

(2) $$\begin{array}{l} {\bar{d}}_{x_{i}}^{in}=\frac{\sum_{j=1}^{\left\|D_{x_{i}}\right\|}wdist\left(x_{i},x_{j}\right)}{\left\|D_{x_{i}}\right\|} \end{array}$$

where $wdist\left(x_{i},x_{j}\right)={\frac{1}{1+e^{-\text{dist}\left(x_{i},x_{j}\right)}}}^{*}\text{dist}\left(x_{i},x_{j}\right)$, dist(*x*_*i*_, *x*_*j*_) is the Euclidean distance of samples *x*_*i*_ and *x*_*j*_, and ||*D*_*x*_*i*__|| is the number of samples in set *D*_*x*_*i*__.

Secondly, let ${\bar{d}}_{x_{i}}^{ex}$ represents the external mean distance which can be obtained with formula (3), and $\bar{d_{c}}$ may be derived with the following sub-steps:

(3) $$\begin{array}{l} {\bar{d}}_{x_{i}}^{ex}=min\left\{\bar{d_{c}}|c\in \left\{1,2,\cdots \;,C\right\},c\neq y_{i}\right\} \end{array}$$

1. For any cluster with label *c*, let *D*_*c*_ = {(*x, y*)|(*x, y*) ∈ *D, y* = *c and y* ≠ *y*_*i*_};
2. For (*x*_*k*_, *y*_*k*_) ∈ *D*_*c*_, calculate dist(*x*_*i*_, *x*_*k*_) which is the Euclidean distance of sample *x*_*i*_ and *x*_*k*_;
3. The weighted distance is $wdist\left(x_{i},x_{k}\right)=\frac{1}{1+e^{-\text{ dist}\left(x_{i},x_{k}\right)}}*\text{dist}\left(x_{i},x_{k}\right)$;
4. Calculate the mean weighted distance of the *c*th cluster by $\bar{d_{c}}=\;\frac{\overset{\left\|D_{c}\right\|}{\underset{j=1}{\sum }}wdist\left(x_{i},x_{k}\right)}{\left\|D_{c}\right\|}$, ||*D*_*c*_|| is the number of samples in set *D*_*c*_.

Finally, the weighted silhouette coefficient of sample *x*_*i*_, i.e., *WSC*_*x*_*i*__, would be obtained with *WSC*_*x*_*i*__ = $\frac{{\bar{d}}_{x_{i}}^{ex}-\;{\bar{d}}_{x_{i}}^{in}}{\max\left\{\overset{ex}{\underset{x_{i}}{\bar{\bar{d}}}},\overset{in}{\underset{x_{i}}{\bar{d}}}\right\}}$.

**Figure 3:**
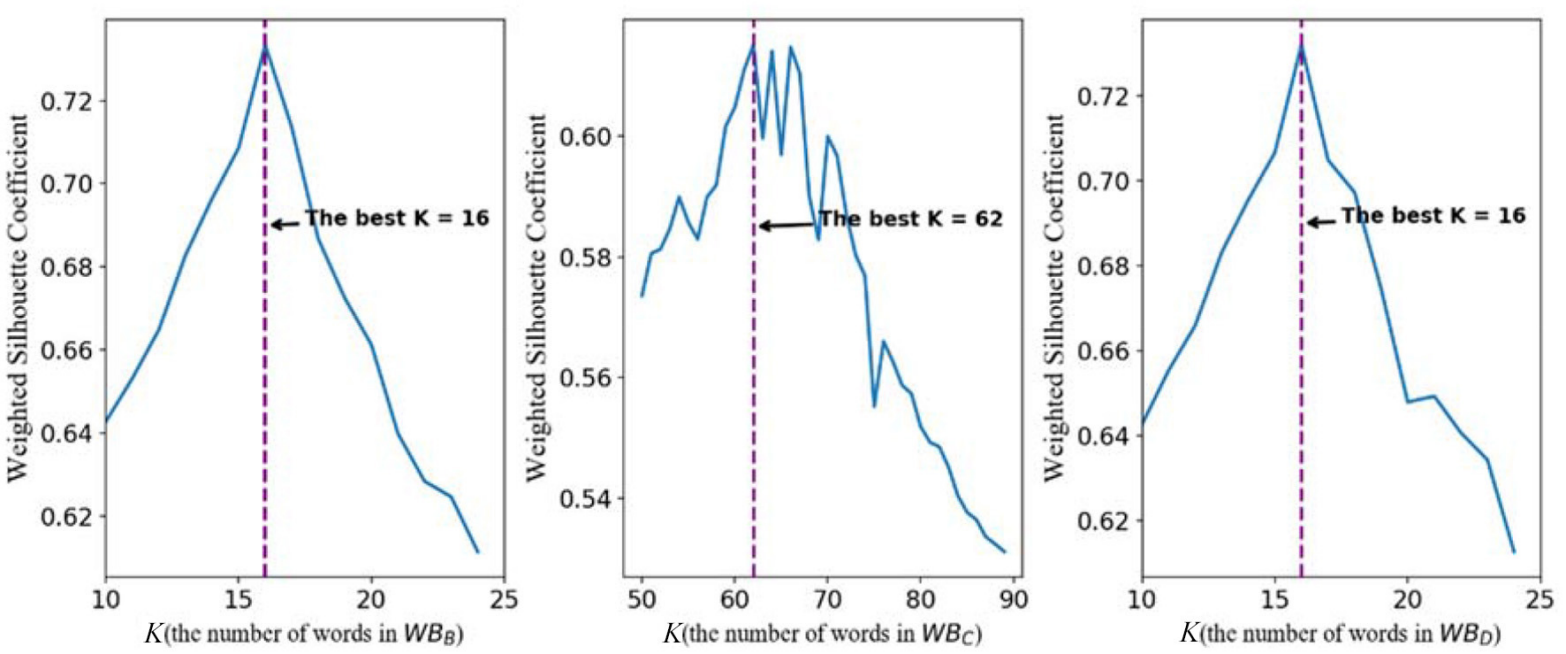
The profile of weighted Silhouette Coefficient and *K*.

For *WB*_*B*_, the line chart of the relationship between weighted silhouette coefficient and *K* is shown in the left subpicture of Figure 3. It is easy to find that, when *K* is 16, the highest weighted silhouette coefficient is achieved. Therefore, the best number of the clustering centers in *WB*_*B*_ is 16. For *WB*_*C*_, it is not difficult to find that the best number of the clustering centers is 62. For the words of the GPCR wordbook *WB*_*D*_, the best number of words is 16 on basis that, when *K* equals 16, the highest weighted silhouette coefficient is achieved.

In summary, the numbers of words of the different wordbooks are shown in Table 1.

- Step 4: Feature extraction based on wordbooks

**Table 1:** The number of words of different wordbooks.

**Wordbook**	**Number of words**
*WB*_*A*_	20
*WB*_*B*_	16
*WB*_*C*_	62
*WB*_*D*_	16

Based on the wordbooks, any GPCR can be represented with a feature vector following the steps below:

1. Encode the GPCR sequence by hydropathy index.
2. Split the encoded sequence into fragments of which the shape is like the shape of each word in the wordbook.
3. Count the number of times each word appears in the sequence.
4. Represent the GPCR in terms of a feature vector with formula (4).

(4) $$\begin{array}{l} G\left(l,C_{l}\right)=\left[f_{1}^{l}{,f}_{2}^{l},\cdots \;,f_{C_{l}}^{l}\right] \end{array}$$

where *l* means the length of a word, *C*_*l*_ is the number of words in the wordbook, and${\;f}_{i}^{l}\;\left(i=1,2,\cdots \;,C_{l}.\right)$ is the frequency of a word in the sequence.

Because of four kinds of wordbooks, any GPCR can be represented as four feature vectors denoted as *G*(1, 20), *G*(2, 16), *G*(3, 62), and *G*(4, 16). Finally, we concatenate the four vectors into a 114-D vector of GPCR listed as follows:

(5) $$\begin{array}{l} G=\left[f_{1}^{1}{,f}_{2}^{1},\cdots \;,f_{20}^{1},f_{1}^{2}{,f}_{2}^{2},\cdots \;,f_{16}^{2},f_{1}^{3},f_{2}^{3},\cdots \;,f_{62}^{3}{,f}_{1}^{4},f_{2}^{4},\cdots \;,f_{16}^{4}\right] \end{array}$$

### Feature Extraction From Drugs

Molecular fingerprint, which is a bit-string representation of molecular structure and property (Eckert and Bajorath, 2007), has demonstrated its effectiveness for the prediction of drug–target interactions in previous studies (Xiao et al., 2013; Hu et al., 2016; Li et al., 2019; Wang et al., 2020b). In this study, we also extract drug features from their molecular fingerprints. A drug's MOL file, which contains information about the chemical structure, can be acquired from the KEGG database (http://www.kegg.jp/kegg/) by using the drug code. Then, the software called OpenBabel (http://openbabel.org/) is used to convert the MOL file into a molecular fingerprint file. OpenBabel can generate multiple output formats: FP2, FP3, FP4, and MACSS. Here the FP2 is a good choice for this study. The FP2 molecular fingerprint is represented by a 256-bit hexadecimal string.

In previous studies, Wang et al. (2020b) and Hu et al. (2016) have confirmed the effectiveness of applying DFT (Jackson, 1996) or DWT (Haar, 1911; Jackson, 1996) on molecular fingerprint, respectively. In this study, we use DFT and DWT for extracting drug features, respectively, and compare the effect of the two kinds of signal processing for predicting the interactions of GPCR—drug pairs later.

For extracting drug features by using DFT, because of the symmetry of the frequency amplitudes of a digital signal, we only choose the first 128 amplitudes to form the drug feature vector *D*_*DFT*_.

(6) $$\begin{array}{l} D_{DFT}=\left[F_{1}{,F}_{2},\cdots \;,F_{128}\right] \end{array}$$

To extract drug features by using DWT, the process should apply single-level discrete 1-D wavelet transform on a digital signal and would reach at two kinds of coefficient sets: one is the set of approximation coefficients which would be considered as useful information, and the other one is the set of detail coefficients which would be recognized as useless noise. Then, we use the set of approximation coefficients to make up the drug feature vector *D*_*DWT*_.

(7) $$\begin{array}{l} D_{DWT}=\left[W_{1},W_{2},\cdots \;,W_{128}\right] \end{array}$$

In the following process, *D*_*DWT*_ or *D*_*DFT*_ is used to represent drugs according to the results of comparative experiments.

Finally, a potential GPCR–drug pair is concatenated to a 242-D feature vector *P* which can be represented by the following formula (8).

(8) $$\begin{array}{l} P=[f_{1}^{A}{,f}_{2}^{A},\cdots {,f}_{20}^{A}{,f}_{1}^{B}{,f}_{2}^{B},\cdots {,f}_{16}^{B},f_{1}^{C},f_{2}^{C},\\ \;\cdots \;,f_{62}^{C}{,f}_{1}^{D},f_{2}^{D},\cdots {,f}_{16}^{D}{,P}^{0}] \end{array}$$

where *P*^0^ means *D*_*DWT*_ or *D*_*DFT*_.

### Feature Extraction by ANN

**Figure 4:**
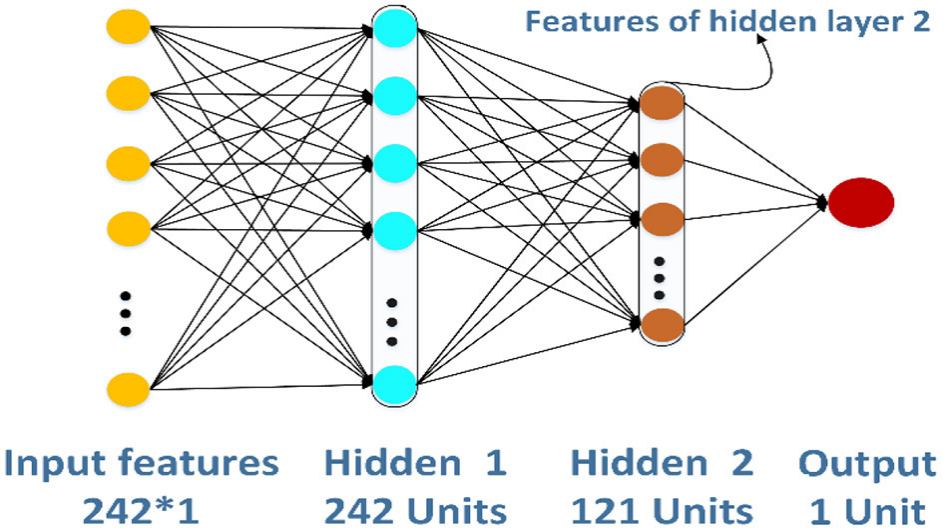
The structure of the artificial neural network for feature extraction.

ANN (Zeng et al., 2019; Wang et al., 2020a; Zhao et al., 2020a,b) is a kind of information processing system based on imitating the structure and function of the brain neural network, which is a complex network structure formed by a large number of interconnected processing units (neurons). In this study, we create a simple ANN model to extract features further, and the structure of ANN is shown in Figure 4. The ANN model has three layers: two layers are hidden layers, and the other layer is an output layer. The 242-D GPCR–drug pair feature vector *P* will input into the model, and the output of hidden layer 2 is intercepted as a new feature.

### Synthetic Minority Oversampling Technique

The Synthetic Minority Oversampling Technique (SMOTE) (Chawla et al., 2002; Blagus and Lusa, 2013; Wang et al., 2019) proposed by Chawla et al. is a very popular oversampling method to solve the problem of imbalance dataset. The basic idea of the SMOTE algorithm is to generate new data from two types of sample data to analyze and simulate a small number of sample sets and add new artificially simulated samples to the dataset. The specific procedure is as follows:

1. Select the *k* nearest neighbors of each sample *x*_*i*_ according to the Euclidean distance between *x*_*i*_ and all samples in the minority class {*x*_1_, *x*_2_, *x*_3_, ⋯, *x*_*m*_} .
2. Set a sampling rate based on the class imbalance ratio *N*, and select *N* samples {*x*_*i*1_, *x*_*i*2_, ⋯, *x*_*iN*_} randomly from *k* nearest neighbors of sample *x*_*i*_.
3. Generate a new sample according to the formula *x*_*new*_ = *x*_*i*_+ α(*x*_*i*_ − *x*_*ij*_); here 1 ≤ *i* ≤ *m*, 1 ≤ *j* ≤ *N* and α represents a random value selected from interval (0, 1).
4. Add new artificially simulated samples to the old dataset and get a new balance dataset.

## Classifier Selection

### Gradient Boosting Decision Tree

The GBDT (Friedman, 2001) is a kind of a boosting algorithm based on classification and regression trees (CART) (Breiman et al., 1984). Because of its strong generalization ability, GBDT has been widely used to be designed as a classifier. GBDT is good at handling lots of kinds of data flexibly, including continuous value and discrete value. The idea of GBDT is to generate multiple weak models iteratively and then add the prediction results of each weak model.

### Random Forest

Random forest (RF) (Breiman, 2001; Song et al., 2017; Cheng and Hu, 2018; Cheng, 2019; Ru et al., 2019; Xu et al., 2019; Lv et al., 2020b) is a kind of bagging algorithm containing many decision trees, which has been widely used in computer science, bioinformatics, and so on. Each tree in the forest is generated by different samples and features. CART is often chosen as the decision tree for RF. When an unknown sample is needed to be classified, each tree will vote, and then RF will count the votes. The unknown sample will be decided to belong to the category with the largest number of votes.

### Support Vector Machines

The support vector machines (SVM) proposed by Vapnik (1995), is a classical machine learning method which has been developed for many years, and its theory has been perfect. It is very popular in bioinformatics, pattern recognition, and so on. The strategy of SVM is to generate the optimal hyperplane based on learning from dataset. There are kinds of kernels in SVM, such as Gaussian radial basis function (RBF), linear kernel, and so on. The most frequently used kernel is RBF.

### Logistic Regression

The logistic regression (LR) (Hosmer and Lemeshow, 1989; Cheng et al., 2019) algorithm used widely in data mining, disease automatic diagnosis, economic prediction, and other fields is one of the most basic and simplest algorithms in machine learning. LR is a kind of a linear classifier which aims at the problem of linear separability. The main idea of using logistic regression to classify is to establish regression formula for classification boundary line according to the training dataset.

## Results

Firstly, DFT and DWT are carried out on molecular fingerprint, respectively, and are evaluated with formula (1) to find the effective one from the two features. It is proved by experiments that applying DWT to extract the features of a drug are better than those of DFT, and then DWT is used to represent drugs. Secondly, an ANN model is established to extract features further, and the prediction performance of GBDT is compared with the different features generated by different layers through cross-validation. Thirdly, a variety of classifiers are applied in the experiments for performance comparison, and GBDT is selected as the default classifier for its good performance. Later, SMOTE algorithm is adopted to balance D92M. Finally, a novel model called BOW-GBDT is proposed and tested with the balance D92M along with the existing models through cross-validation and an independent test. According to the result, BOW-GBDT has a better generalization ability.

### Effect of Different Feature Representations of Drugs

**Figure 5:**
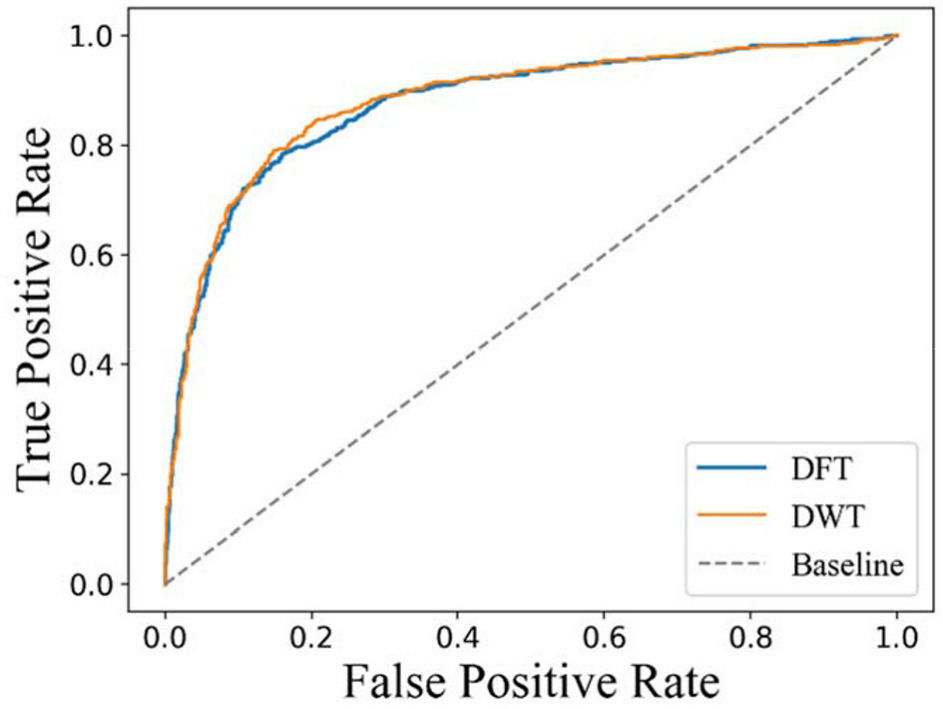
Receiver operating characteristic curves of 10-fold cross-validation on D92M.

In a previous work, carrying out DFT or DWT on molecular fingerprint had been demonstrated to be an effective feature extraction method for drugs. However, there is no experimental comparison between DFT and DWT. In this section, 10-fold cross-validation is carried on D92M while representing drugs with DFT or DWT, respectively. The results about ROC curves are shown in Figure 5. It is clear that the AUC with a value of 0.890 and ROC of DWT is better than that of DFT (whose AUC value is 0.876). Therefore, we use DWT as the default method to extract features from drugs in this study.

### Effect of Different Features Generated by ANN Models

**Figure 6:**
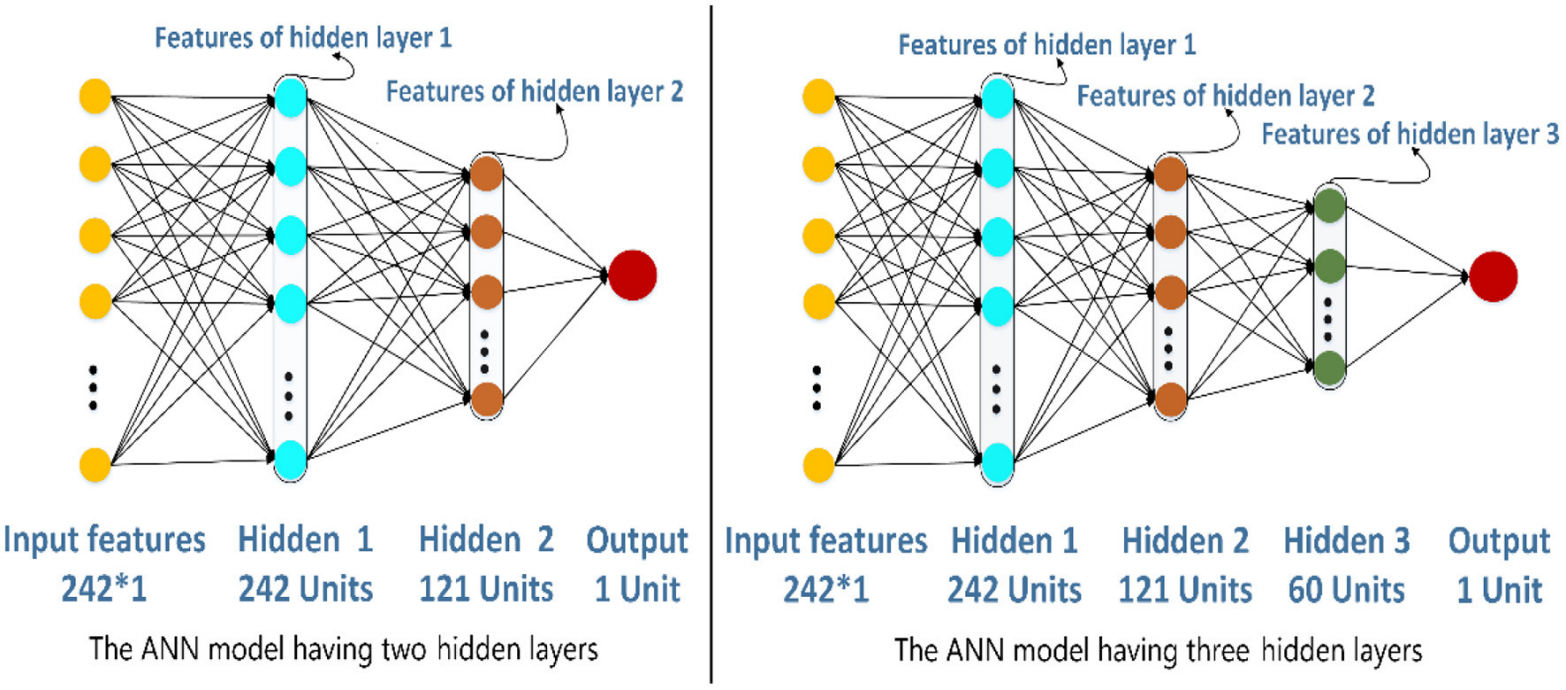
The different artificial neural network model.

The structure of ANN is very flexible. In this section, we would decide the number of hidden layers in the ANN model. To be simple, the number of units of hidden layer 1 is 242, and the next hidden layer has half the number of units compared with the previous hidden layer. There are two different structures of the ANN model in Figure 6. The left one has two hidden layers whose number of units are 242 and 121, respectively. The right one has three hidden layers whose number of units are 242, 121, and 60, respectively. In this paper, the ANN models are built and trained using Tensorflow, which is a popular Python software package. The hyperparameters including learning rate, epochs, and batch size of the two models are set as 0.01, 100, and 128, respectively. The activation function of the hidden layers and the output layers are LeakyReLU and Sigmoid separately.

**Figure 7:**
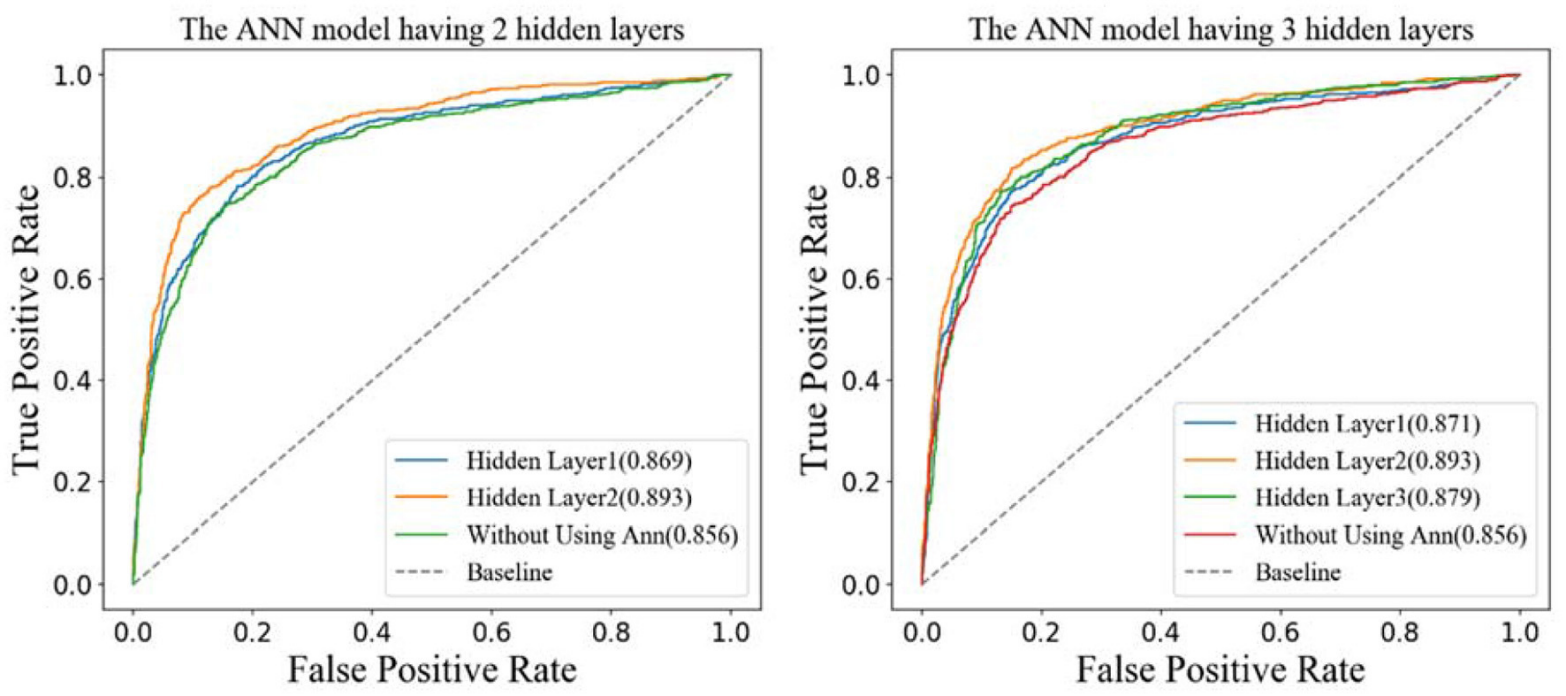
Receiver operating characteristic curves of 10-fold cross-validation on D92M.

According to the structures of the different ANN models, the features generated by different hidden layers would be extracted from the two models separately, and the results of the ROC curves are shown in Figure 7. From the figure on the left, we can see that the AUC (0.893) of the features generated by hidden layer 2 is bigger than the one generated by hidden layer 1 (0.869) in the ANN model having two hidden layers. The results in the figure on the right show that the AUC of hidden layer 2 is bigger than the one of hidden layer 3 and hidden layer 1 in the ANN model having three hidden layers. What is more, the AUC of hidden layer 2 of the two models is close to 0.893. Considering that the ANN model having two hidden layers is simpler than the one having three hidden layers, we adopt the ANN having two hidden layers in this research and the features generated by hidden layer 2 as the final features.

### Choose a Better Classifier

For a binary classification problem, the machine learning algorithm (Larrañaga et al., 2006) is very important to some extent. The knowledge learned by different algorithms from the same dataset may be very different, and the generalization ability is also different. In this section, we compare the performance of different algorithms by carrying out leave-one-out cross-validation on D92M. The algorithms that we adopt and the result values of Sn, Sp, Acc, Str, and MCC are listed in Table 2. Compared with the results of different machine learning algorithms, the Sn, Acc, Str, and MCC of GBDT gain most of good performance as marked with an italic font in the last line of Table 2. Therefore, we determine to adopt GBDT as the default algorithm to build prediction models.

**Table 2:** The results of different algorithms.

**Algorithms**	**Sn (%)**	***Sp* (%)**	**Acc (%)**	**Str (%)**	**Matthews correlation coefficient**
RF	70.6	*94.2*	86.1	82.4	0.68
SVM	63.8	93.8	83.5	78.8	0.62
LR	52.9	87.6	75.8	70.3	0.44
GBDT	*76.1*	93.9	*87.8*	*85.0*	*0.72*

*Italic mean that they are the best scores compared with other methods*.

### The Effect of SMOTE Algorithm

The dataset D92M containing 635 interactive GPCR–drug pairs and 1,225 non-interactive pairs is an imbalance dataset. The previous work (Yamanishi et al., 2008; He et al., 2010; Xiao et al., 2013; Hu et al., 2016; Wang et al., 2020b) did not deal with the imbalanced problem of dataset. In this study, we use the SMOTE algorithm to deal with the imbalance dataset and get a new balance dataset. Then, the balance dataset and the imbalance dataset are input into GBDT over leave-one-out cross-validation, respectively. The results of Acc, MCC, Sn, Sp, and Str are listed in Table 3.

**Table 3:** The results of the model with the SMOTE algorithm or without.

**Datasets**	**Sn (%)**	**Sp (%)**	**Acc (%)**	**Str (%)**	**Matthews correlation coefficient**
Imbalance dataset	76.1	*93.9*	87.8	85.0	0.72
Balance dataset	*79.5*	93.1	*88.5*	*86.3*	*0.74*

*Italic mean that they are the best scores compared with other methods*.

As can be seen in Table 3, the Sn, Acc, Str, and MCC values increase by 3.4, 0.7, and 1.3% and 0.02, respectively. The results show that the SMOTE algorithm can improve the performance of GBDT. Therefore, the SMOTE algorithm is used to deal with the imbalance dataset D92M.

### Comparison of Other Methods

In order to confirm the performance of our model called BOW-GBDT, we test them on D92M and Check390, respectively, and compare it with existing methods, such as IGPCR-Drug, OET-KNN, QuickRBF, and so on. The results of the different methods on D92M over leave-one-out cross-validation are shown in Table 4, along with those of other eight methods listed in Xiao et al. (2013). As shown in the table, the DWKNN has the biggest value of Sn, and the SP, Acc, Str, and MCC values of BOW-GBDT are higher than those of other methods. This result confirms the good performance of the proposed method.

**Table 4:** Performance of different methods tested with leave-one-out cross-validation.

**Method**	**Sn (%)**	**Sp (%)**	**Acc (%)**	**Str (%)**	**Matthews correlation coefficient**
IGPCR-Drug	78.3	91.4	86.9	84.9	0.71
OET-KNN	77.8	88.7	85.0	83.3	0.67
QuickRBF	74.8	92.4	86.4	83.6	0.69
SVM	74.2	92.7	86.4	83.6	0.69
RF	76.5	92.9	87.3	84.7	0.71
RF + PPP	79.7	92.8	88.3	86.3	0.73
DWKNN	*81.4*	84.7	83.6	83.1	0.64
DWKNN(Ensemble)	81.1	87.1	85.1	84.1	0.67
BOW-GBDT	79.5	*93.1*	*88.5*	*86.3*	*0.74*

*Italic mean that they are the best scores compared with other methods*.

Though BOW-GBDT achieves a good result in leave-one-out cross-validation, the generalization ability is more important for a machine learning model. We use the SMOTE algorithm to balance the D92M and generate a new dataset. With the new dataset as training dataset and Check390 as the independent test, the results of the other eight methods mentioned in Xiao et al. (2013) are also listed in Table 5. From this table, we can notice that the proposed model BOW-GBDT has a better generalization ability. Like the result in Table 4, BOW-GBDT has the highest values of Sp, Acc, Str, and MCC besides Sn. Compared with other state-of-the-art methods, the Acc of BOW-GBDT is 3.9% higher than the second one, the Sp is 5.8% higher than the second one, the Str is 2.1% higher than the second one, and the MCC is 0.07 higher than the second one. This result demonstrates that BOW-GBDT is a good model for predicting the GPCR–drug interaction.

**Table 5:** The results of different methods over independent test dataset Check390.

**Method**	**Sn (%)**	**Sp (%)**	**Acc (%)**	**Str (%)**	**Matthews correlation coefficient**	**Threshold**
IGPCR-drug	80.8	66.9	71.6	73.9	0.45	N/A
OET-KNN	67.7	84.2	78.7	76.9	0.52	0.5
QuickRBF	76.2	77.7	77.2	77.6	0.52	0.45
SVM	76.2	78.9	78.0	77.6	0.53	0.42
RF	78.5	78.1	78.2	78.3	0.54	0.51
RF + PPP	83.1	79.6	80.8	81.3	0.6	0.51
DWKNN	*83.9*	80.0	81.3	81.9	0.61	0.5
DWKNN (ensemble)	83.1	82.7	82.8	82.9	0.63	0.5
BOW-GBDT	80.0	*90.0*	*86.7*	*85.0*	*0.70*	0.5

*Italic mean that they are the best scores compared with other methods*.

## Conclusions

In this paper, the authors proposed a new method for predicting the interaction between GPCR and drug. In terms of representation GPCR, a BOW model was used to extract features from GPCR sequences. For the representation of drugs, the DWT method was applied for the reason that DWT can have a better prediction performance than DFT. The highlight of this study is that the ANN model was introduced to extract more effective features by automatically learning from the original features. What is more, a popular and powerful oversampling algorithm called SMOTE was applied to balance the training dataset. According to the results on the D92M over leave-one-out cross-validation and the testing dataset Check390, the proposed method has a better generalization ability. By the way, the structure of the ANN model is very flexible, and it is hard to find the best model containing how many hidden layers and the units in every layer. Actually, this method gets a good performance for predicting the GPCR–drug pair interaction by using a simple ANN model containing two hidden layers, yet there is still room to be improved in the future.

**Figure 8:**
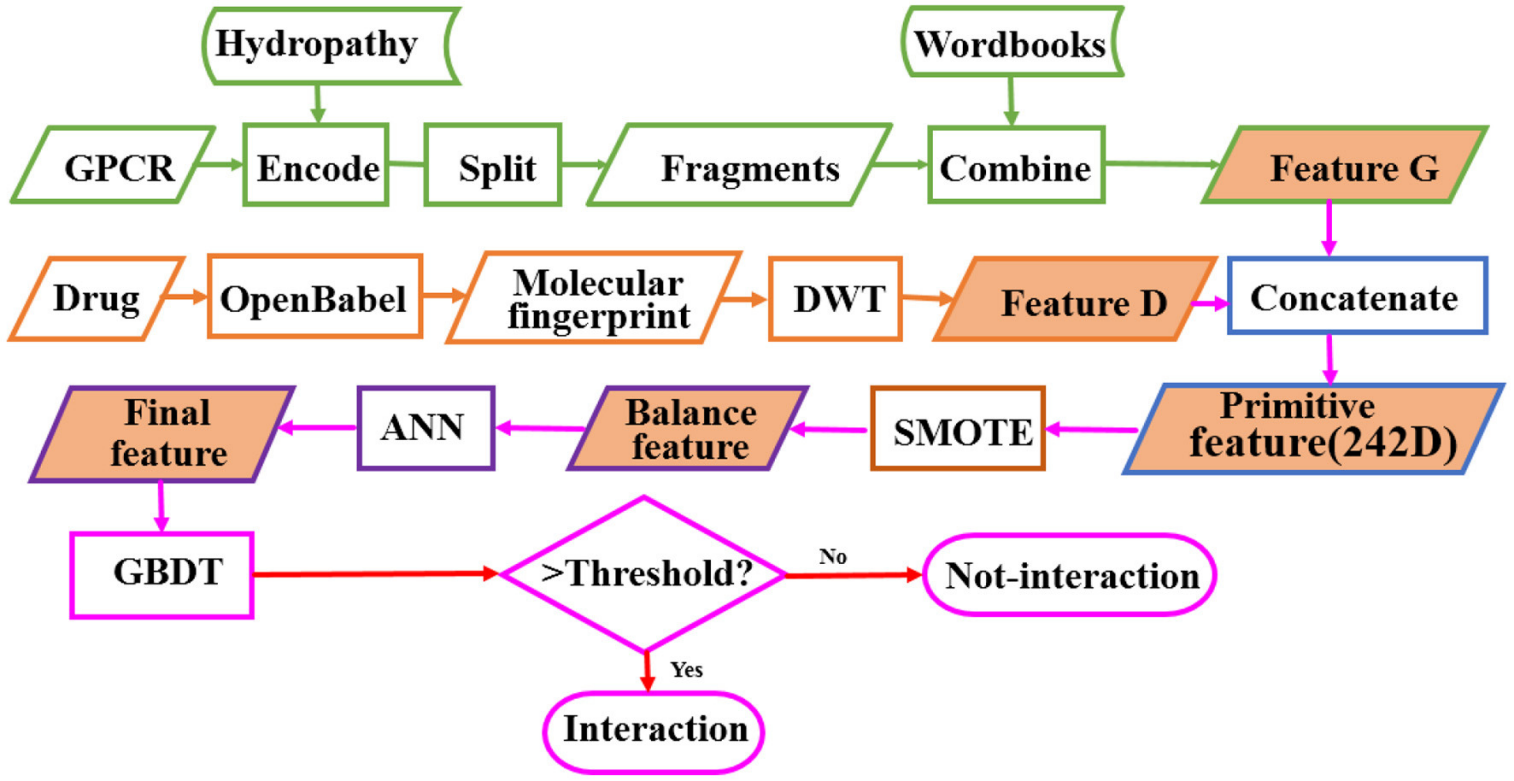
The framework of BOW-GBDT.

GPCRs are involved in many physiological processes such as photosensitivity, regulation of the immune system, regulation of the autonomic nervous system, regulation of behavior and emotion, and so on. They are the most import drug targets in modern medicine. The research on identifying the interaction between GPCRs and drugs is of great importance for the discovery of GPCR-related drugs. In order to solve the problem of high cost and low efficiency of high-throughput experimental methods, we develop a model called BOW-GBDT based on GBDT algorithm for predicting the interaction between GPCR and drug. The proposed framework of BOW-GBDT can be summarized as shown in Figure 8. The boxes marked with a green border show the representation process for GPCR and tawny for drug. Although BOW-GBDT has better performance as compared to other methods when it is tested in dataset Check390, it should still be tested in other datasets to evaluate it further.

## Data Availability Statement

The original contributions presented in the study are included in the article/supplementary material, further inquiries can be directed to the corresponding author/s.

## Author Contributions

WQ conceived and designed the experiments. ZL performed the extraction of features, model construction, model training, and evaluation. YH and JJ analyzed the data and implemented the classifiers. ZL and WQ drafted the manuscript. XX supervised this project and revised the manuscript. All authors read and approved the final manuscript.

## Conflict of Interest

The authors declare that the research was conducted in the absence of any commercial or financial relationships that could be construed as a potential conflict of interest.
